# ERAS—Enhanced Recovery After Surgery: The ERAS Society Story

**DOI:** 10.1002/wjs.70378

**Published:** 2026-04-29

**Authors:** Olle Ljungqvist, Mary Brindle, Martin Hubner, Gregg Nelson, Ulf Gustafsson

**Affiliations:** ^1^ Department of Surgery School of Medical Sciences Örebro University Örebro Sweden; ^2^ Department of Molecular Medicine & Surgery Karolinska Institutet Stockholm Sweden; ^3^ Department of Community Health Sciences Cumming School of Medicine University of Calgary Calgary Alberta Canada; ^4^ Ariadne Labs Harvard TH Chan School of Public Health and Brigham and Women's Hospital Harvard University Boston Massachusetts USA; ^5^ Department of Visceral Surgery University Hospital CHUV University of Lausanne (UNIL) Lausannois Switzerland; ^6^ Department of Obstetrics & Gynecology Cumming School of Medicine University of Calgary Calgary Alberta Canada; ^7^ Ariadne Labs Brigham and Women's Hospital Harvard T.H. Chan School of Public Health Boston Massachusetts USA; ^8^ Department of Clinical Sciences at Danderyd Hospital Karolinska Institutet Stockholm Sweden; ^9^ Department of Pelvic Cancer, Division of Coloproctology Center for Digestive Diseases Karolinska University Hospital Stockholm Sweden

## 1 Introduction

In the 1990s a group of US cardiac surgeons presented a bundled care process that shortened length of stay and called this “Fast Track surgery” [1]. This approach was further developed and popularized by a colorectal surgeon Henrik Kehlet. Kehlet showed that patients were able to be discharged 2 days after colectomy [2] if treated with a selection of specific care elements in a multimodal approach to recovery [3]. This was a revolution at a time when it was common for colorectal patients to stay in hospital for 2 weeks or longer. The improvements that were apparent with this new model of care was the inspiration to the initiation of Enhanced Recovery After Surgery (ERAS); a concept of care that has since spread across the world and rolled out to many different types of surgery. This paper tells the story of the development of ERAS from inside the ERAS Society.

## 2 The ERAS Study Group

The Enhanced Recovery After Surgery (ERAS) study group was formed in 2001 [4]. The idea was to spread and scale the multimodal approach through international collaboration. The challenge was to broaden the ideas from Fast Track by reviewing the literature for all elements of perioperative care that could contribute to recovery after colonic surgery. The group worked for almost 10 years developing the concept of ERAS. During this time, the interest in ERAS grew as the group published a range of papers [4]. A consensus statement on a protocol for care for colonic surgery was published in 2005 that gained a lot of attention and is viewed as a landmark paper in the ERAS story [5]. These early papers have also formed the basis for the mission of the ERAS Society in 2010.

## 3 The Early Insights to Enhance Recovery

The first published ERAS protocol identified multiple elements of care that enhanced recovery. Despite the ERAS study group members agreeing on the best practices of the ERAS protocol, when the members who created the protocol reviewed their own patient care, they found their actual daily practices were quite different from what they thought they were doing. These clinicians were not using the full ERAS protocol and were delivering care quite differently from the care they knew should be best. The ERAS study group decided that formal implementation of the protocol was required. For this purpose, a common database was constructed to collect demographics, process and care data and outcomes. When the first data was reviewed, key observations were made, most notably that compliance was lower than the group thought, and the care that was being delivered was not what they had believed. Even when clinicians were convinced that care was being provided according to their protocol they found it was only partially being done. A large case series published shortly after this data collection process had begun showed that complications and length of stay related closely to how well the protocol was followed [6]. The inflection point at which best outcomes could be achieved appeared to be around 70% compliance to the pre‐ and intraoperative care elements. The ongoing challenge recognized was to ensure that every patient received the right care every time and for every element of care. A protocol was not enough to reach the very short hospital stays that the Danish group had achieved [7]. It was also clear that without continuous audit, there was poor recognition of performance failings. The core value of the audit is as a mechanism to advance sustainability. A team from the Netherlands showed that with structured implementation using well proven methodology, units trained to use the ERAS protocol could reduce hospital stay by 30%–50% within a year [7]. However, the focus of their work was on initial implementation and data collection and feedback focused on comparing outcomes before and after implementation. Follow up showed that compliance dropped and outcomes regressed to near baseline levels after the project finished [8]. These findings suggest that a new way of working to keep continuous track of ERAS compliance and outcomes would be necessary for sustainability.

During these early years a few small, randomized trials were performed, and a meta‐analysis showed that more ERAS elements as opposed to fewer had marked impact not only on length of stay but resulted in an almost 50% reduction in complications for colonic surgery [9]. After almost 10 years of using ERAS in their practice, the original ERAS Study Group recognized that there was an opportunity and need to share the learnings of ERAS beyond isolated teams. They decided to form the ERAS Society, creating a global cross‐professional perioperative Society unlike any other.

## 4 The Formation of the ERAS Society and the ERAS Project Plan

The pillars of the ERAS Society were based on the knowledge gained and helped form the mission of the Society (Table 1).

**TABLE 1:** The mission statement of the ERAS Society is to enhance recovery after surgery and develop perioperative care and to improve recovery through research, audit, education and implementation of evidence based practice.

Mission statements	ERAS Society initiatives
Evidence based practice	Development of guidelines covering all care elements shown to improve recovery
Global spread and scale	Making ERAS accessible to hospitals and perioperative teams across the globe through open access guidelines and free educational initiatives.
Audit	Encouraging global ERAS teams to pursue audit and feedback as a core part of any ERAS program. The ERAS Interactive Audit System (EIAS) was developed as the Society's endorsed audit system to record demographics, care processes and outcomes for all patients continuously
Implementation	ERAS leaders will ensure that principles and guidelines can be translated into actions by supporting protocol and implementation tool development and advising implementation programs based on a foundation of continuous audit and feedback (i.e., EIAS) with an ultimate goal of achieving sustained and meaningful compliance
Research	Expanding the scope and quality of the ERAS research mandate including the use of the EIAS database as a cornerstone of high‐quality research
Education	Foster education that supports the new way of teams working, publish educational material including textbooks

The issue encountered most frequently was not uncertainty about best practice ‐ the knowledge that formed the ERAS guidelines was often well supported by accepted evidence in the literature. The main barriers to best care relate to implementation. Standardized ways of training hospitals to drive the change of practice partnered with a reliable audit and feedback process offered a pathway to ensure that the best knowledge was translated into bedside care.

The nature of enhanced recovery practice dictated that any intervention that aimed to improve the delivery of ERAS must be multidisciplinary and multiprofessional. Most hospital staff work in silos and have little if any insight about what happens to the patient before or after they come under their care. One of the core goals of ERAS is to break down the clinical silos in patient care so all members of the care team would be aware of their intersecting roles in the delivery of care in its entirety.

The early experience from a common database has tremendous benefits for research. The data collected across countries has been validated in three countries to accurately reflect medical records [10, 11, 12] and allows for the findings previously based on small, single center experiences to be explored using large numbers and multiple different contexts [6, 13]. Using the same database applied to research to drive ERAS implementation across hospitals allows for real‐world data to inform ongoing care.

When looking to devise a strategy for global, standardized implementation, the academic and non‐profit ERAS Society was challenged. The requirements to develop, maintain and advance a standardized dataset and provide deep, meaningful implementation support comes at a significant financial and human resource cost.

Various suggestions and approaches for financing were considered by the Society but, in the end, it was decided that there was a need for a commercial partner who could lead a resource‐heavy and ambitious goal to develop a platform for global ERAS implementation including maintenance of a robust database.

In 2009 and the formal formation of the ERAS Society, a not for profit medical Society based in Sweden, was finalized in 2010 and an agreement signed with the startup for profit company Encare AB supported by the incubators of the Karolinska Institutet and STING, in Stockholm Sweden.

The development and maintenance of this partnership have required careful consideration to maintain the scientific integrity and independence of the Society while capitalizing on the opportunities to achieve and sustain the highest levels of care through robust ERAS implementation and maintain a powerful global database collecting information not simply on outcomes but also implementation. The ERAS Society constantly revisits the relationship with Encare in order to maintain its role as a source for trusted science and its democratic ambitions to make ERAS accessible to any perioperative team across the globe.

## 5 The Spread of ERAS and How it Has Developed

ERAS started with colorectal surgery but colleagues from a range of different specialties have recognized that the benefits of ERAS can translate across specialties. The ERAS Society has grown over the years with the formation of sub‐societies and guidelines for a range of surgical domains and procedures. ERAS guidelines are multiprofessional and multimodal and the scope of the perioperative journey. As such, their structure is unique and the approach to creating robust, and trusted guidelines has required the development of an “ERAS approach” to guideline generation. This “guideline for guidelines” based on GRADE methodology, is now the backbone for all new ERAS® guidelines [14]. The ERAS Society maintains a repository of open access guidelines available to the global community (www.erassociety.org) with a range that spans a large representation of elective surgical practice, and recently also emergency surgery and trauma. The value of ERAS compliance, first noted in the early days of enhanced recovery, has been reenforced in the findings of research across several surgical specialties: with each unit increase in ERAS compliance, there is a corresponding decrease in both length of stay and complications (a so‐called dose response relationship) [6, 15, 16].

Across different specialties, ERAS delivers shorter stay by an average of 2 days, a reduction in complications by 30% and substantial savings for the cost of care, Table 2 [17, 18]. Long term follow‐up suggests a survival benefit from ERAS [19, 20], likely related to avoidance of postoperative complications [20, 21].

**TABLE 2:** Key outcomes reported from implementation of ERAS protocols in major surgery.

Main outcome	Effect
Complications	Reduced by approximately 30%–50%
Need for postoperative hospital stay	Reduced by ≈30%, average 2 days
Return on investment for ERAS implementation	Up to 7 times
Effect on long term survival	Improved or equal

*Note:* The magnitude of the improvements is related to the type of surgery and the degree of improved compliance to the guideline element achieved.

**FIGURE 1:**
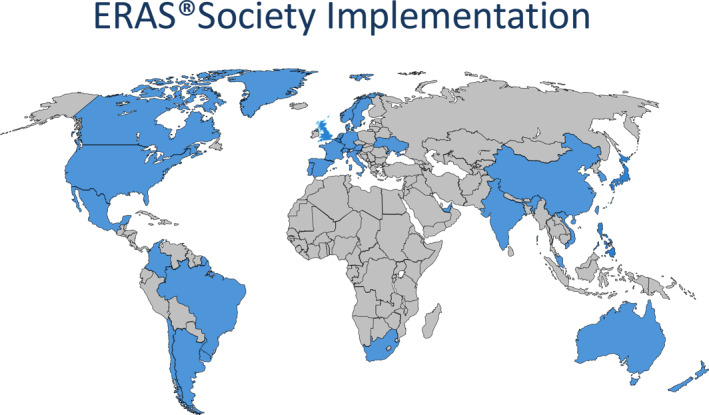
Shows the countries in blue where the ERAS Society has been conducting implementation programs.

One of the core functions of the ERAS Society has been to build a community of researchers, clinicians and health system leaders from across the globe. The Society has held annual international congresses since 2012, with the exception of years during the COVID pandemic. The Society has also aligned with many specialty societies, such as the International Surgical Society and others, and in specialty congresses around the world ERAS is now a standard feature session. Within the Society there is a growing network of ERAS trained hospitals and many national chapters that build national expertise and hold national or regional conferences (Figure 1).

Both through free educational workshops and open webinars as well as, more intensively, through its relationship with Encare, the Society has supported training in hospitals across the globe. Today the ERAS Society has supported ERAS implementation training in hospitals in more than 30 countries world‐wide. The Society also aligned with World Journal of Surgery as their official journal much because of the wide reach that the journal has especially in low‐ and middle‐income countries where the Society has a special focus.

The ERAS philosophy has diffused broadly. In some countries, societies for perioperative care and enhanced recovery separate from the ERAS Society have been formed. These societies also help spread the word of Enhanced Recovery/Fast Track and run their own versions and trainings of enhanced recovery. The ERAS Society resources remain open for these organizations, and the Society continues to learn and grow from the experiences of Enhanced Recovery both inside and outside of the Society's structures.

## 6 Challenges and the Future

A future focus of the ERAS Society is to support the adoption of ERAS globally and enhance the capacity for the Society to support and lead research across the world. The unique database is a goldmine for clinical outcomes research that has considerable unmined potential [10, 22, 23, 24] There have been multinational data published but more can be done.

An ongoing challenge is that most clinical units involved in surgery today claim they “do ERAS”. While some may in fact have successful ERAS in place, on closer inspection most units are not truly practicing ERAS or producing the results that would be anticipated if ERAS was truly, meaningfully adopted [25, 26]. The experience from more than a decade of training hospitals and auditing their perioperative care most units, even with dedicated ERAS programs, have between 40% and 60% compliance to ERAS guidelines and national data on length of stay shows twice that of true ERAS care [27, 28]. With focused interventions to improve compliance above 70%–80%, the clinical benefits show up.

The stringent and timely production of guidelines proves another challenge alongside the lack of data for many surgeries as mentioned above. Guidelines have a lifespan as data evolves and systems change. Guidelines need to both be developed with rigor but also updated every few years to keep up with the best evidence. The impacts of COVID impacted the Society, and clinical demands continue to challenge academic clinicians who lead and develop these guidelines.

Despite the obvious benefits for patients including fewer complications and shorter length of hospital stay, ERAS is far from being adopted universally and much work is still needed. No hospital has full control over its care processes which remains a major hurdle to improve practice, and the continuously raised barriers to data‐sharing between sites and countries without compromising individual integrity represents yet another obstacle to development.

## 7 Conclusions

After more than 20 years of Enhanced Recovery, we have seen concepts that were once challenging and controversial become accepted as the standard for best perioperative care. Beyond developing a strategy and guidelines for providing best perioperative care, the ERAS Society now aims to deepen its impact in places where ERAS adoption remains poor‐supporting the democratization of ERAS knowledge, powerful implementation, and robust data to inform the future of Enhanced Recovery care.

## Author Contributions

**Olle Ljungqvist:** conceptualization, writing – original draft, methodology, writing – review and editing, formal analysis, supervision, project administration, validation, visualization. **Mary Brindle:** validation, writing – review and editing, conceptualization, formal analysis. **Martin Hubner:** conceptualization, writing – review and editing, formal analysis, validation. **Gregg Nelson:** conceptualization, writing – review and editing, formal analysis, validation. **Ulf Gustafsson:** conceptualization, writing – review and editing, formal analysis, validation.

## Funding

The authors have nothing to report.

## Conflicts of Interest

Olle Ljungqvist is a co‐founder and past chairman of the ERAS Society, currently serving as the Ambassador. On behalf of the ERAS Society founders, Dr Ljungqvist founded and own shares in Encare AB, Sweden. Dr Ljungqvist reports personal fees for travel and lecturing from Abbot and Encare AB. Dr. Nelson reports personal fees (non‐recurring) from Intuitive, Integra Life Sciences, and Smith & Nephew outside the submitted work, and is Chair of the Scientific Committee of the ERAS Society. Dr. Brindle is the Secretary General of the ERAS Society. Dr Hubner is the Chairman of the Guideline Committee of the ERAS Society. Dr Gustafsson is the Chairman of the Executive committee, of the ERAS Society.

## Data Availability

The authors have nothing to report.
